# Cardiovascular/Stroke Risk Stratification in Parkinson’s Disease Patients Using Atherosclerosis Pathway and Artificial Intelligence Paradigm: A Systematic Review

**DOI:** 10.3390/metabo12040312

**Published:** 2022-03-31

**Authors:** Jasjit S. Suri, Sudip Paul, Maheshrao A. Maindarkar, Anudeep Puvvula, Sanjay Saxena, Luca Saba, Monika Turk, John R. Laird, Narendra N. Khanna, Klaudija Viskovic, Inder M. Singh, Mannudeep Kalra, Padukode R. Krishnan, Amer Johri, Kosmas I. Paraskevas

**Affiliations:** 1 Stroke Monitoring and Diagnostic Division, AtheroPoint™, Roseville, CA 95661, USA; dr.anudeeppuvvula@gmail.com (A.P.); drindersingh1@gmail.com (I.M.S.); 2 Department of Biomedical Engineering, North Eastern Hill University, Shillong 793022, India; sudip.paul.bhu@gmail.com (S.P.); mahesh.nehu.333@gmail.com (M.A.M.); 3 Annu’s Hospitals for Skin & Diabetes, Gudur 524101, India; 4 Department of CSE, International Institute of Information Technology, Bhuneshwar 751003, India; sanjay@iiit-bh.ac.in; 5 Department of Radiology, University of Cagliari, 09121 Cagliari, Italy; lucasabamd@gmail.com; 6 Deparment of Neurology, University Medical Centre Maribor, 1262 Maribor, Slovenia; monika.turk84@gmail.com; 7 Heart and Vascular Institute, Adventist Health St. Helena, St. Helena, CA 94574, USA; lairdjr@ah.org; 8 Department of Cardiology, Indraprastha APOLLO Hospitals, New Delhi 110001, India; drnnkhanna@gmail.com; 9 Department of Radiology and Ultrasound, University Hospital for Infectious Diseases, 10000 Zagreb, Croatia; klaudija.viskovic@bfm.hr; 10 Department of Radiology, Harvard Medical School, Boston, MA 02115, USA; mkalra@mgh.harvard.edu; 11 Neurology Department, Fortis Hospital, Bangalore 560010, India; pudukode.krishnan@fortisheakthcare.com; 12 Department of Medicine, Division of Cardiology, Queen’s University, Kingston, ON K7L 3N6, Canada; amerschedule@gmail.com; 13 Department of Vascular Surgery, Central Clinic of Athens, 106 80 Athens, Greece; paraskevask@hotmail.com

**Keywords:** Parkinson’s disease, cardiac autonomic dysfunction, cardiovascular disease, stroke, artificial intelligence, deep learning, machine learning, recommendations

## Abstract

Parkinson’s disease (PD) is a severe, incurable, and costly condition leading to heart failure. The link between PD and cardiovascular disease (CVD) is not available, leading to controversies and poor prognosis. Artificial Intelligence (AI) has already shown promise for CVD/stroke risk stratification. However, due to a lack of sample size, comorbidity, insufficient validation, clinical examination, and a lack of big data configuration, there have been *no well-explained bias-free AI investigations* to establish the CVD/Stroke risk stratification in the PD framework. The study has two objectives: (i) to establish a solid link between PD and CVD/stroke; and (ii) to use the AI paradigm to examine a well-defined CVD/stroke risk stratification in the PD framework. The PRISMA search strategy selected **223** studies for CVD/stroke risk, of which **54** and **44** studies were related to the link between PD-CVD, and PD-stroke, respectively, **59** studies for joint PD-CVD-Stroke framework, and **66** studies were only for the early PD diagnosis without CVD/stroke link. Sequential biological links were used for establishing the hypothesis. For AI design, PD risk factors as covariates along with CVD/stroke as the gold standard were used for predicting the CVD/stroke risk. The most fundamental cause of CVD/stroke damage due to PD is *cardiac* *autonomic dysfunction due to neurodegeneration that leads to heart failure and its edema,* and this validated our hypothesis. Finally, we present the novel AI solutions for CVD/stroke risk prediction in the PD framework. The study also recommends strategies for removing the bias in AI for CVD/stroke risk prediction using the PD framework.

## 1. Introduction

Parkinson’s disease (PD) is a neurological disorder that causes a progressive loss of coordination and motor difficulties. The condition is named after James Parkinson, a British surgeon who published the first explanation of it in 1817 [1]. PD is triggered by the loss and malfunctioning of neurons (nerve cells) in the *substantia nigra*, a portion of the brain. PD is characterized by difficulties with dopaminergic neurons, which are brain cells that connect with other neurons by producing a signaling substance called dopamine, also known as a neurotransmitter [2,3,4]. The cost of the treatment and control of the PD is expensive [5]. This treatment cost of PD is more, as depicted in various studies [1,6]. Further, more numbers of PD cases were seen in western countries as compared to Asian countries [7,8].

Stroke is one of the leading causes of mortality and severe and long-term disability across the world [9,10]. Hemorrhagic and ischemic strokes are the two forms of stroke. The first is produced by blood clots obstructing brain arteries, whereas the second is caused by vascular rupture [11,12]. According to the World Health Organization (WHO), stroke is the second leading cause of death and the third major cause of morbidity and mortality each year, accounting for 6.2 million fatalities in the world [9,13].

Although PD has repeatedly been linked to an increased risk of all-cause of death in several epidemiologic studies, the data on the link between PD and stroke are mixed [14,15]. According to a few studies [16,17,18], PD is associated with a greater risk of myocardial infarction and stroke-related death [19,20], with a hazard ratio ranging from 1.5 to 3.6. Furthermore, new research suggests that PD is linked to vascular risk factors, including diabetes and hypertension [21,22]. Meanwhile, other research has indicated that PD patients had a decreased risk of stroke and have a lower frequency of vascular risk factors [23]. Diabetes and hypertension are also risk factors for ischemic stroke; the link between PD and stroke may be complicated [24,25].

Complex motor damage can result when PD and CVD are linked to the development and phenotype of PD [24,26]. Capillary segmentation and associated damage to the capillary network in diverse areas of the brain are caused by vascular abnormalities [27]. The *substantia nigra*, the midfrontal cortex, and the basal nuclei of the brain are all affected by leukoaraiosis [28,29]. All of these factors reduce the impact of antiparkinsonian therapy on motor and cognitive abilities [30].

**Figure 1:**
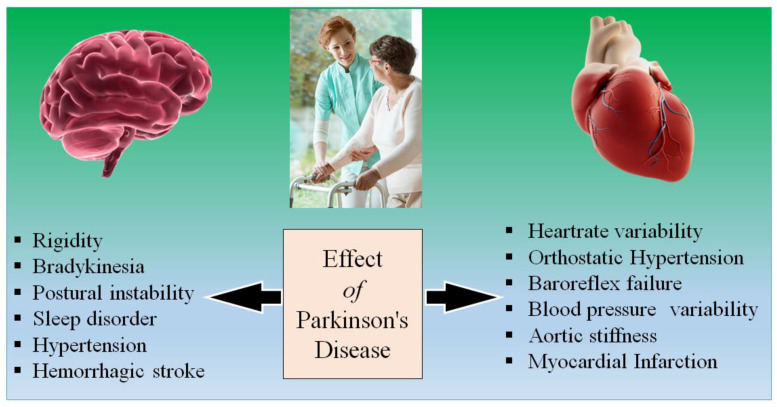
Long-term effect of Parkinson’s disease on the brain and heart.

Patients having PD are always on the higher risk side of the heart and brain functioning abnormalities [31,32,33]. Heart and brain relative functionality were explained in many articles [32,34,35]. The alpha-synuclein can trigger abnormalities in the functioning heart and brain. Figure 1 represents the effect of PD on the severity of brain and heart functioning. There are two key reasons why the automatic coordination of the heart system is affected in people with PD. First, the regions of the brain that control this system frequently contain Lewy bodies and have experienced neurodegeneration [36,37]. Furthermore, Lewy body-like accumulations and neurodegeneration have a direct impact on the autonomic nervous system. This implies that when the heart and carotid artery baroreceptors detect a reduction in blood pressure and try to send a signal to the heart and blood vessels to raise blood pressure, the message may not be received [38,39]. Due to autonomic nervous system malfunction, this causes neurogenic orthostatic hypotension (nOH) or dips in blood pressure when standing. There are no drugs that can restore the autonomic nervous system to treat nOH [40,41]. When it comes to the cardiac symptoms of PD, the focus is usually on nOH, which then creates changes in heart rate, which is another cardiac impact in PD [42]. Heart rate variability [43,44], which is a measure of the variation in the time interval between heartbeats, was found to be higher in patients who eventually developed PD than in those who did not, suggesting that cardiac autonomic dysfunction could be an early non-motor symptom of the disease [45,46].

A few of the studies explain that persons with PD exhibit particular electrocardiographic characteristics. These characteristics include a longer PR interval and probably a longer QTc interval, which refers to parts of the cardiac tracing that are longer than normal [47,48].

Artificial intelligence (AI)-based solutions aid in the automated assessment of COVID-19 severity in patients by using lung images as inputs, removing the need for human intervention. Several CVD risk assessment applications employing carotid ultrasound imaging have also benefited from AI-based methods [49,50,51,52,53]. As a result, it may be conceivable to use these AI-based methods to effectively tackle PD-CVD and PD-Stroke or hemorrhage brain disorders when performing the patient risk evaluation.

AI-based methods have played a vital role recently in computer-aided diagnosis [54,55,56], especially in the detection and classification of several diseases [57,58]. It was only recently that the application of machine learning (ML) has dominated the field of medical imaging such as diabetes [59,60], cancer such as thyroid [61,62], liver [58] prostate [54,63], skin, ovarian [55,64], and now more in non-invasive vascular screening [65], risk characterization using coronary, and carotid angiography [66,67]. Several medical imaging modalities are available for imaging, such as magnetic resonance imaging (MRI) [68,69], computed tomography (CT) [70], ultrasound (US) [71], particularly CT for lung imaging depicting COVID-19 symptoms and their lesions. The deep learning (DL) algorithm was used to segment the COVID-19 lungs and further to detect the lesion in CT lung scans [68,72,73]. ML models have been used in predicting PD as it contains a variety of the motor symptoms features (called covariates) available in PD datasets [74,75,76]. We, therefore, hypothesize that ML/DL systems can be adopted for CVD/stroke risk prediction in PD patients, hence evolving a design strategy would benefit in the future.

The objective of this review is to understand the severity of heart failure and stroke in PD patients, the risk factors of CVD, the clinical linking between PD with heart and brain, and its effect vice-versa. More important is to understand the role of AI in the risk stratification of CVD/stroke in PD patients. Since machine learning and deep learning solutions help in establishing the early risk assessment of PD patients, this is being demonstrated for the characterization of CVD, ischemic, and hemorrhage stroke in PD. Lastly, a brief note of the PD in a COVID-19 affected environment help in looking at and accessing the current problems faced in disease management as well as the pathophysiology of the PD [77].

## 2. Methods

**Figure 2:**
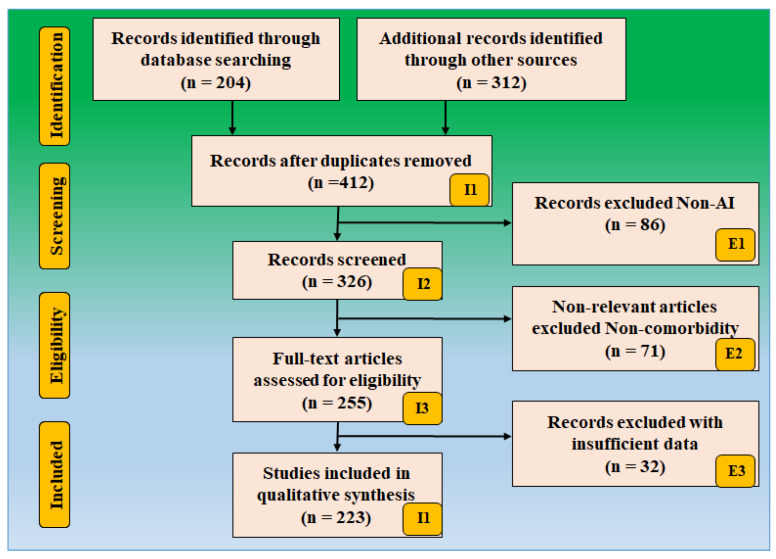
Search strategy based on the PRISMA model.

The search approach was based on the PRISMA paradigm shown in Figure 2. PubMed and Google Scholar are two major databases that were used to identify and screen relevant papers using keywords such as “Cardiovascular disease,” “Stroke,” “CVD,” “Stroke and CVD,” “Parkinson’s disease and CVD,” “Parkinson disease and Stroke,” “carotid imaging,” “Parkinson disease and artificial intelligence,” “atherosclerotic tissue classification and characterization,” “plaque tissue characterization in Parkinson disease,” “artificial intelligence,” “Parkinson disease and COVID-19,” “atherosclerosis and Parkinson disease.” A total of 204 records were identified through database searching, and 326 items were found through other sources. This was reduced to 412 articles after quality custom criteria such as time and relevance. A total of 326 papers were reviewed for inclusion in this review. The three exclusion criteria were (i) studies not related, (ii) non-relevant articles, and (iii) having insufficient data. This excluded 86, 71, and 32 studies were shown as E1, E2, and E3, leading to the final selection of 223 studies.

These studies, which are in category (i), are studies that are not related. These studies either do not have AI or do not show risk stratification of CVD/stroke in PD patients. There were 86 studies that we removed from the selection process, shown as E1 in the PRISMA model. Non-relevant studies are ones that are not in the field of view of PD-CVD-stroke. They were not focused on the PD-CVD-stroke area. Our focus in this study was only on those papers where PD was related to CVD and stroke. If the studies showed an association between PD and Diabetes, we did not take that into consideration. There were 71 studies in this category, shown as E2 in the PRISMA model.

These insufficient data studies were the studies that did not have enough information to be selected for consideration in our analysis. These studies did not show a link between PD and CVD or PD and stroke. No such discussions were attempted. There was no consideration between PD and CVD risk parameters such as laboratory-based biomarkers, which include low-density lipoprotein, high-density lipoprotein, estimated glomerular filtration rate, erythrocyte sedimentation rate, and triglycerides. Further, they did not have enough AI or CVD, or stroke attributes to be selected for analysis. These AI attributes can be the architecture used for CVD/stroke risk stratification. These AI attributes can be solo deep learning models or deep hybrid learning, or neural network parameters for CVD/stroke risk stratification. We found 32 studies that had insufficient datasets shown as E3 in the PRISMA model. The complete research article screening process is shown in Figure 2.

## 3. The Relationship between PD and Combined Heart and Brain Diseases

PD is still the most prevalent neurodegenerative disorder, with symptoms and signs such as tremors, bradykinesia, stiffness, and involuntary movements [78,79,80]. Pathological factors responsible for aberrant protein aggregation development, alteration of protein elimination routes, oxidative stress, neuroinflammation, mitochondrial damage, and genetic abnormalities all contribute to the formation of the clinical complexity in PD [81,82]. Heart failure, coronary artery disease, and PD are the main cause of cardiac autonomic dysfunction, heart failure, sudden death, and edema [83]. PD will increase the likelihood of developing dementia disorders and is linked with a high rate of morbidity and death [17,84]. To control disease progression, various methods were used, such as stem cell therapy, gene therapy, exercise, dopaminergic medications, and non-dopaminergic drugs. In the therapy of PD, nutrition and surgical treatment are crucial [85,86].

### 3.1. The Relationship between PD and Atherosclerosis Leading to CVD

**Figure 3:**
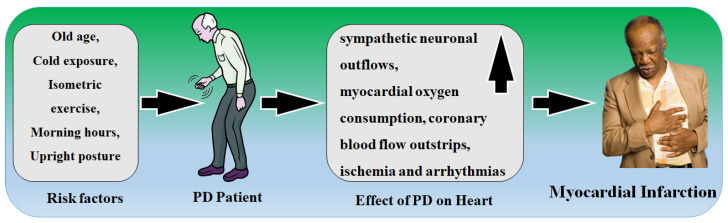
A risk factor in PD patients responsible for myocardial infarction.

The accumulation of plaque in the inner lining of an artery causes atherosclerosis or thickness or blockage of the coronary arteries [87]. The autonomic nervous system (ANS) regulates several systems, including cardiovascular regulation baroreceptors or blood pressure valves, which are found in the heart and the carotid artery [88]. When the baroreceptors detect a change in the blood pressure, a signal is transmitted to certain brain locations [89]. The ANS then transmits impulses to the heart, which regulates heart rate and cardiac output [90]. Signals are also transmitted to the arteries, which cause them to contract and regulate blood pressure [91]. Both CVD and PD have a strong link to diabetes, advanced age, and male gender. Glucose metabolism, cellular stress, lipid metabolism, and inflammation are all affected by genetic, environmental, and biological variables [92,93]. Stroke is the most prevalent medical issue among the elderly [94]. However, research on the link between PD and stroke has yielded mixed results [24,95]. Strokes, such as cerebral infarction, frequently coincide with PD pathology, according to autopsy studies, and individuals clinically diagnosed with PD frequently have a concurrent cerebral infarction [11]. Figure 3 shows the risk factor in PD patients responsible for myocardial infarction.

It is observed that PD patients exposed to a cold environment, isometric activity, the morning hours, upright posture, and advanced age have enhanced sympathetic neuronal discharges, which leads to increased myocardial oxygen demand [27,96]. Normally, autonomic modifications aid homeostasis; however, in the presence of a separate pathological condition, coronary arterial stenosis increases oxygen consumption given by coronary blood flow, which exceeds the supply, resulting in ischemia and arrhythmias [97,98].

The effect of metabolic syndrome is always linked to a group of cardiovascular risk factors that include abdominal obesity, elevated blood pressure (EBP), dyslipidemia, and dysglycemia, all of which are linked to the development of CVD and a higher chance of death from CVD and other causes [99].

**Figure 4:**
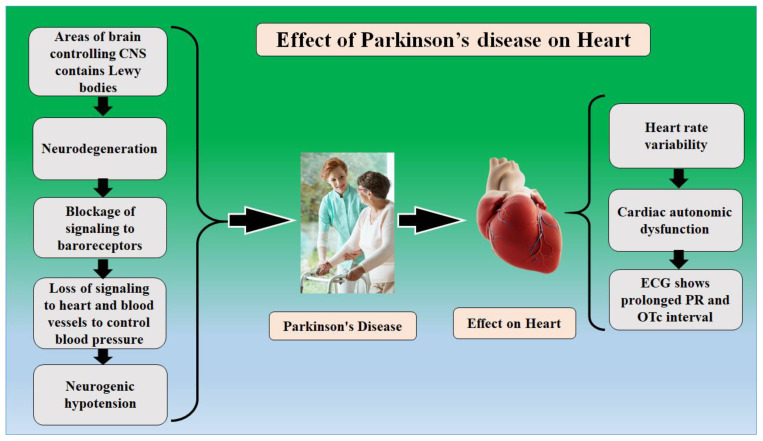
The relation between Parkinson’s disease and Heart.

The ANS is part of the peripheral nervous system, which is a network of nerves that runs throughout the body [100]. Respiration, heart function, blood pressure, digestion, urine, sexual performance, pupillary response, and many other processes are controlled by the ANS [101]. The parasympathetic nervous system and the sympathetic nervous system are two subsystems of the ANS [102]. Most main organs are regulated by both the parasympathetic and sympathetic nervous systems [103]. They frequently have opposing effects, with the sympathetic nervous system stimulating a system while the nervous system is regulating it [104]. Figure 4 shows the relationship between PD and autonomic dysfunctions.

Table 1 represents various attributes that relate to the link between PD and CVD. Orthostatic hypotension and cardiac abnormalities are the most prevalent medical issue among the elderly.

### 3.2. The Relationship between Parkinson’s Disease with the Brain

The second leading cause of death in PD patients is stroke [115,116]. It is also the sixth leading cause of long-term impairment [117]. Hemorrhagic stroke has been identified as a primary cause of morbidity and mortality [118]. When cerebral blood flow is disrupted, neuroinflammatory cascades are activated, which can affect brain metabolism and lead to neuronal death [119]. Carotid stenosis occurs when the carotid arteries narrow, preventing smoother blood flow [23]. The sudden rupture of a blood artery within the brain causes obstructions in hemorrhagic stroke [120]. Stroke severely damages the brain and its cognitive functions [121]. Cerebral infarction is intimately linked to PD due to cerebrovascular and neurodegenerative disorders coinciding [122]. Although levodopa causes OH and raised homocysteine, which may increase the risk of stroke, it remains the most effective and essential symptomatic therapy for many people with PD [123].

L-dopa is still the first line and gold-standard treatment for PD [124]. The use of L-dopa has been proven to raise homocysteine levels in the blood [125]. The conversion of S-adenosyl methionine to S-Adenosyl-L-homocysteine and then homocysteine is linked to the pathophysiological process of O-methylation of L-dopa to 3-O-methyldopa [47,110]. The PD patients under the L-dopa medication and homocysteine are at higher risk of leading towards cardiovascular problems [126]. Ventricular arrhythmia is the most dangerous adverse effect of levodopa in patients, which is uncommon to develop in a healthy heart but a concern in individuals with myocardial irritability or ischemia [127]. Patients who have had ventricular ectopic activity in the past should be treated with care and electrocardiographically monitored [128].

**Figure 5:**
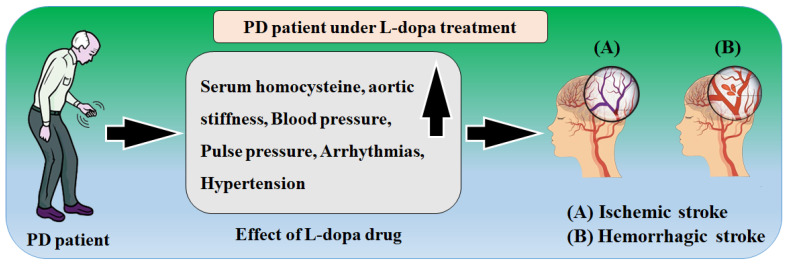
The relationship between L-dopa and stroke.

The dosage of levodopa should be progressively raised; if ectopic activity is detected, the medicine should be withdrawn or combined with an antiarrhythmic treatment, the most reasonable of which is adrenergic blockers [33]. Other antiarrhythmic medications may be effective if these are contraindicated. Orthostatic hypotension, which is more prevalent than symptomatic, should be monitored with regular blood pressure readings in the standing posture, and exercising should be avoided [129]. The link between L-dopa with stroke is shown in Figure 5. The most prevalent side effect of long-term levodopa treatment is movement issues (motor fluctuations) [130]. Within 5 to 10 years, the majority of persons who use levodopa experience these issues [131]. The impact of wear and tear is the most common kind of levodopa-related motor fluctuations.

When the effects of a single dose of levodopa do not last as long as they did before, this is known as a wearing-off time [132]. As the medicine’s effects wear off, control of motor symptoms deteriorates, and symptoms do not improve until the next dosage of levodopa is administered [133]. Based on the time of each pharmaceutical administration, these motor variations are straightforward to anticipate [134]. Dyskinesia is involuntary motions that are typically jerky or writhing that will affect the head, neck, limbs, and legs, as well as other areas of the body [135]. As a result of variable dopamine levels in the brain, “on” and “off” phases occur without warning [136]. The symptoms are comparable to those experienced as a result of the wearing-off effect, but they are more difficult to predict and manage. The patient may freeze during an “off” period, which comes unexpectedly over seconds or minutes [137]. During the “on” phases, on the other hand, uncontrolled movements may occur [138]. Treatment of PD by using L-dopa may modify the plasma metabolome implicated in phenylalanine and tyrosine metabolism, reducing bile acid increases in Parkinson’s disease [139]. Table 2 indicates the relationship between PD and stroke; most of the studies mention observations related to the risk associated with PD, which is stroke, traumatic brain injury, and heartrate variability.

### 3.3. The Relationship between PD and Combined CVD and Stroke

**Figure 6:**
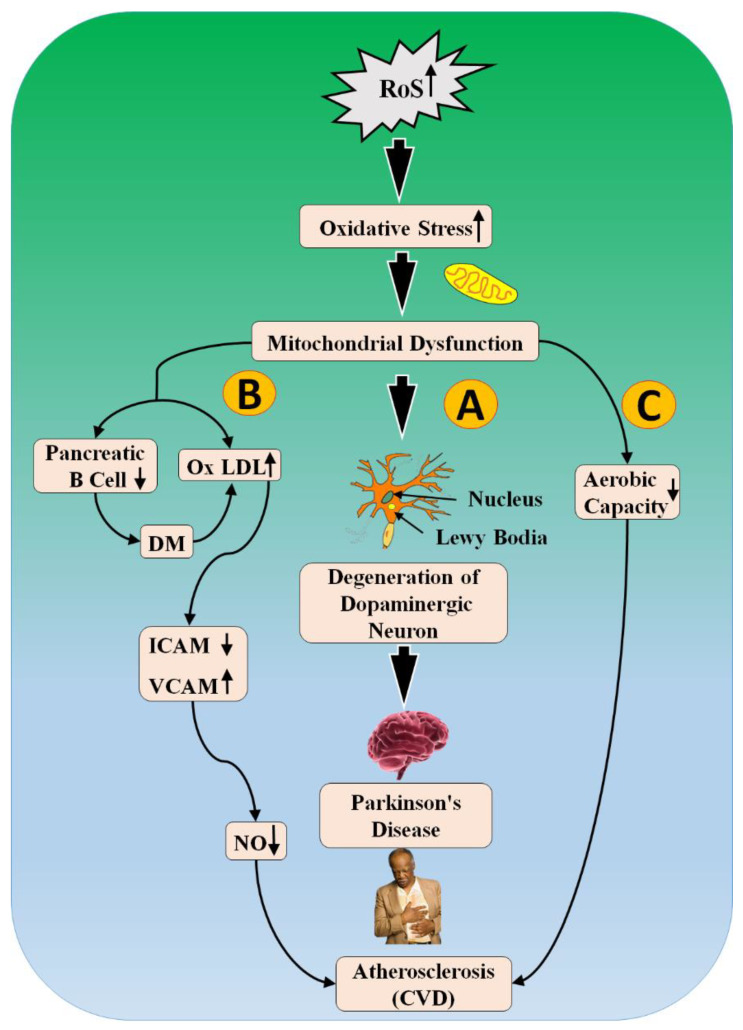
The biological link between PD and CVD. RoS: reactive oxides stress, ICAM: intercellular adhesion molecule, VCAM: vascular cell adhesion molecule, DM: Diabetes mellitus, NO: nitric oxide, OxLDL: oxidation of low-density lipoprotein; Up Arrow: depicts increase; Down Arrow: depicts decrease.

Many references have shown that the most important contributing factor behind the development of PD leading to CVD is oxidative stress [144,145]. Figure 6 explains the biological relationship between PD and CVD. Excessive production of reactive oxygen species (ROS) encourages mitochondrial dysfunction [146]. However, it also triggers the process of atherosclerosis in various mechanisms, as supported by Yu et al. [147] and Bennett et al. [148], which explains that there is a positive relation between mitochondrial DNA damage and the formation of atherosclerosis. Further, mitochondrial dysfunction also leads to damage to the heart via three different paths, as represented in Figure 6.

Path (A) explains the role of oxidative stress as a central step for selective degeneration of dopaminergic neurons in *substantial nigra* of the brain [149]. This damage results in three cardinal symptoms of PD such as resting tremor, rigidity, and loss of balance [150]. In path (B), oxidative stress results in damage to pancreatic beta cells and increased formation of Oxidation of low-density lipoprotein (OxLDL).

This further causes dysfunction of endothelial cells in blood vessels [151]. Damaged endothelial cells increase the adhesive property by increasing levels of intercellular adhesion molecule (ICAM) and vascular cell adhesion molecule (VCAM) [152]. These cause a decrease in levels of (nitric oxide) NO and promote the formation of atherosclerotic plaque [153]. Additionally, path (C) shows the relationship between mitochondrial dysfunction that decreases the aerobic capacity, which is a strong risk factor for CVD [154,155]. This shared pathogenesis between PD and CVD is because of excessive ROS and mitochondrial dysfunction [156].

### 3.4. The Role of the Shared Gene in Parkinson’s with CVD and Stroke

A deficiency in the PRKAG2 gene causes a right bundle branch block or anterior hemiblock in these people [92]. Hyperhomocysteinemia trinomial has a relationship between PD and an increased risk of ischemic stroke [140]. High levels of homocysteine have been linked to an increased risk of stroke, coronary artery disease, and dementia, and levodopa medication has been linked to an increase in blood homocysteine levels [140]. Homocysteine promotes the generation of free radicals and inflammation [129]. Genetic investigations, on the other hand, have demonstrated that PD and stroke share pathophysiology [157]. The gene phosphatase and tensing homolog deleted on chromosome 10 (PTEN) was found to control the formation of ROS in both PD and stroke models [158]. DJ-1 (PARK 7) is also a gene associated with premature hereditary PD [18].

The autonomic and, eventually, blood pressure and heart rate adaptations that accompany acute cardiovascular stresses daily are supported by the baroreflex system [159]. As a result, altered neuronal cardiovascular responses might result from poor baroreflex function (i.e., lower sensitivity or gain) [160].

PD affects both the parasympathetic and sympathetic branches of the autonomic nervous system, which are both controlled by the baroreflex system, and a thorough knowledge of this important process is required [161]. In PD, arterial stiffness, a decreased proportion of C1 neurons, and stimulation of non-C1 synapses, central alpha-synuclein accumulation, cardiac autonomic nerve impingement, reduced muscular sympathetic nerve activity, and lower norepinephrine release might all impact baroreflex function [162].

Hyperglycemia, insulin resistance, advanced glucose end degradation products, reactive oxygen radicals, sphingolipid accumulation, oxidized LDL cholesterol buildup, and an elevation in C-reactive protein are all symptoms of this condition [31]. The progression of PD, coronary heart disease, diabetes, and high blood pressure is induced by these pathways [85]. Folded protein aggregates, alteration of protein disposal routes, mitochondrial damage, oxidative stress, excitotoxicity, neuroinflammation, and abnormalities are all essential pathogenic factors in Parkinson’s that influence patient hospitalization [112]. Blood pressure fluctuations can occur even in the early stages of PD due to autonomic nervous system malfunction [163]. Orthostatic hypotension, postprandial hypotension, nocturnal hypertension, and supine hypertension are all symptoms of autonomic nervous system failure [164].

In Section 3 we have seen the relationship of PD with CVD, brain, and combined CVD with the brain. The biological link between PD and CVD with the brain. The effect of the PD on CVD and stroke and hypothesized that the most fundamental cause of CVD/stroke damage due to PD is cardiac autonomic dysfunction due to neurodegeneration, which leads to heart failure and its edema.

In PD, there is always risk associated with CVD and stroke; hence early risk stratification is very important to avoid mortality [159]. AI systems are already implemented to predict the risk of CVD, stroke, and Parkinson’s but individually; hence there is scope to develop an integrated AI model for early risk stratification of CVD and stroke complications in PD patients. The further section explains the role of AI-based systems for CVD/stroke risk assessment and possible architecture for PD patients.

## 4. Machine Learning-Based System for CVD/Stroke Risk Assessment for PD Patients

Machine learning has been a powerful paradigm since it uses a knowledge-based model for building the training system. Recently, there have been attempts to design ML systems covering several applications such as diabetes [59,60,165], neonatology [166], gene [167,168], coronary artery disease risk stratification [169,170], carotid plaque classification [171], cancer imaging such as thyroid [61,172,173], breast [174], ovarian [64,175], prostate [63,176], etc. The second key benefit of ML is its ability to handle the nonlinearity between the combination of risk factors (or covariates) and the gold standard. This was recently shown for cardiovascular risk stratification [30,177,178,179,180].

The HDL algorithm also plays an important role in handling the nonlinear feature extraction. HDL consists of the concatenation of two solo deep learning models, or sometimes HDL, also referred to as a concatenation of solo DL with an ML model. HDL has shown to have superior performance compared to solo DL and solo ML models [181,182,183,184,185].

**Figure 7:**
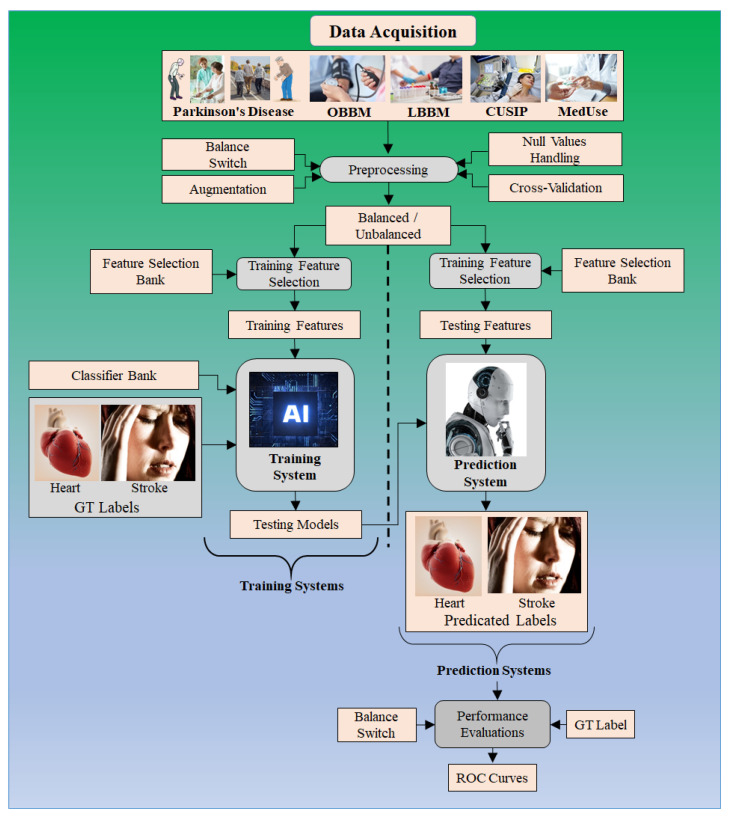
ML model for CVD/stroke risk assessment using Parkinson’s disease.

These risk factors are the amalgamation of (i) PD covariates; (ii) conventional laboratory and office-based covariates; (iii) atherosclerosis covariates; and (iv) current medication uses as covariates. The gold standard is either heart failure (cardiovascular events) or a stroke (cerebrovascular events). Figure 7 shows the AI model for CVD/stroke risk assessment using PD.

The conventional covariates are the risk factors which are a combination of office-based biomarkers (OBBM), laboratory-based biomarkers (LBBM), carotid image-based phenotypes (CUSIP), and medication usage (MedUSE). CUSIP is the image-based phenotype derived using angiographic screening of the blood vessels [65].

Due to cost reasons, one can prefer non-invasive imaging of the carotid arteries for atherosclerosis imaging [71] with noise-reduced imaging [186,187]. Segmentation of the carotid walls helps in the identification of the plaque built-up [66,188]. The review demonstrates how PD leads to the worsening of CVD and stroke in a gradually sequential activity. We suggest a method for using AI to aid in the detection of CVD/stroke risk stratification in the PD framework. Table 3, Table 4 and Table 5 represent studies that use AI for the detection of CVD, stroke, and PD, respectively.

## 5. Critical Discussions

### 5.1. Principal Findings

The first study is the symptomatic observations of CVD and stroke risk stratification in the environment of PD and further investigates the risk factors and gold standards for PD patients having CVD and stroke risk stratification. The effects of PD on the brain and heart are widely known. The review demonstrates how PD leads to the worsening of CVD and stroke in a gradual sequential activity. We suggest a method for using AI to aid in the detection of CVD/stroke risk stratification in the PD framework. As a result, in addition to PD screening, as a low-cost approach, we can use gold standard coronary artery scans as covariates for the stroke risk stratification to prevent worsening of CVD/stroke conditions in PD patients. Effective monitoring of these patients can be conducted with the help of an AI-based model, and long-term consequences for the patients can be avoided.

Machine learning and deep learning aid in the more accurate risk assessment of CVD and stroke in the PD framework. The model may be taught in such a way that it requires no human involvement and produces speedy results. In today’s healthcare systems, this shows to be a revolution, especially in the CVD and stroke risk stratification in the PD framework. Clinicians can use the vascular and cerebrovascular data-based outcome of the AI model to counsel PD patients and advise them on the risk stratification of Cardiovascular/stroke that comes with it.

Our research shows that PD patients, particularly those with high-risk CVD and stroke, should choose CVD and stroke risk assessment methodologies. Patients with PD benefit from carotid imaging for the diagnosis of heart conditions. Ultrasound-based imaging techniques have been shown to be the most convenient for carotid imaging, according to our findings. Furthermore, AI-based algorithms are the ideal choice for the detection of CVD/stroke risk stratification in the PD framework. All of these indicators should thus be followed to diagnose and treat the condition as soon as possible.

### 5.2. Benchmarking

After an analysis of different studies, there were a few research articles that discussed the connection between PD with CVD, PD with stroke using OBBM, LBBM, CUSIP, and MedUse. Few of the articles explain the role of AI in the diagnosis and risk stratification of CVD, stroke, and PD but separately. Nevertheless, no single article explains the stroke and CVD risk stratification in PD patients by using AI. Table 6 represents a comparative analysis of the different studies.

Bikias et al. [199] mentioned that freezing of gait (FoG) is a mobility issue that affects people with PD in their latter stages. Despite the PD patient’s best efforts, it causes the inability to walk, leading to a loss of coordination that increases the risk of falls and accidents and hurts the PD patient’s quality of life. Stress, emotional stimulation, and multitasking have all been linked to the onset of FoG episodes, with the patient’s functioning and self-confidence worsening with time. By examining inertial measuring unit data, this study provides a non-invasive way of detecting FoG events. Deep FoG achieves 83%/88% sensitivity/specificity for leave one out cross-validation and 86.5%/90% sensitivity/specificity for 10-fold CV schemes, respectively, according to experimental data.

Another study by Reva et al. [195] explained the first AI-based algorithms capable of reliably and effectively measuring collateral flow in patients with AIS described in this paper. This automated technique for assessing collateral filling might help clinical decision-making for determining reperfusion-eligible patients by streamlining clinical workflow, reducing bias, and assisting in clinical decision-making. In patients with major artery blockage acute ischemic stroke who receive reperfusion treatment, collateral circulation is linked to a better functional prognosis. Because of the complicated neuro vasculature, assessing collateral flow may be time-consuming, subjective, and challenging. Bermejo et al. [105] commented that Parkinson’s autonomic dysfunction is a prevalent non-motor symptom. The majority of dysautonomic disorders are caused by changes in the autonomic nervous system’s peripheral nerves, which include both the sympathetic and parasympathetic nervous systems. Cardiovascular impairment is common in patients with PD due to the degradation of sympathetic nerve cells and neurons. This unpleasant side effect restricts the therapeutic use of L-dopa in elderly patients with PD and can increase the frequency of hospitalizations. As a result, defining the cardiac characteristics associated with PD is critical for monitoring the heart status in parkinsonians. Furthermore, the article by Pramanik et al. [200] described two recent decision forest algorithms, Systematically Developed Forest and Decision Forest by Penalizing Attributes, with the widely used Random Forest to create three distinct Parkinson’s detection schemes with the least number of decision trees. The proposed decision forests, SysFor, and forest, as well as the widely used Random Forest, have been used as Parkinson’s detectors. The suggested Parkinson’s detection approach uses incremental decision trees and training examples, which is a unique contribution to the area of Parkinson’s detection. Mouridsen et al. [196] showed non-contrast computed tomography (CT), and magnetic resonance imaging (MRI) can be used to differentiate between ischemic and hemorrhagic stroke, which is difficult to identify solely on clinical symptoms. Although the sensitivity of MRI is higher in the acute situation, hypodensity on CT and DWI hyperintensity on MRI identify permanently injured tissue. Angiographic and perfusion imaging sequences can detect a major artery obstruction and, in combination with perfusion imaging, can identify individuals who should be treated with endovascular treatment.

In conclusion, neither study, to our knowledge, has offered such additional insight into multiple approaches to illnesses that are needed for CVD/stroke risk stratification in the PD framework.

### 5.3. A Special Note on PD-Stroke Hypothesis

Vascular Parkinsonism is caused by a stroke that affects the *substantia nigra* of the basal ganglia [206]. Damage is mostly caused by a loss of blood circulation to certain areas of the brain, as it is with other strokes [207]. Small vessel strokes are the most common type of stroke associated with Parkinsonism because they are not usually fatal. Small vascular strokes can be diagnosed with diagnostic methods such as a CT scan or an MRI of the brain [208]. The symptoms of vascular Parkinsonism are usually brought on by a series of minor strokes [209]. Small artery strokes can sometimes lead to vascular dementia, which is a kind of dementia [210]. As a result, patients with vascular Parkinsonism are more likely to develop vascular dementia. The most hazardous side effect of anti-PD drug levodopa in patients is atrial arrhythmias, which is uncommon in a healthy heart but a problem in those with myocardial instability or hypoxia [211].

### 5.4. A Special Note on PD-CVD Hypothesis

Due to the failure of ANS, cardiomyopathies are relatively uncommon in people who have PD [212]. These individuals have an increase in left ventricular mass, left ventricular pressure, left atrial volume, concentric remodeling, and diastolic dysfunction; such condition can lead to heart failure, which may develop at a later stage [213]. PD is linked to atherosclerotic risk factors, including hypertension and diabetes. These individuals are more likely to develop coronary artery disease [106]. PD is associated with an increase in mortality due to vascular disease, and sudden cardiac death is an uncommon complication of PD [214]. Conduction defects, hypertonia, ventricular arrhythmias (due to the medications employed), and cardiomyopathies are all the causes of abrupt cardiac death [215]. These variations in blood pressure can be detected in the early stages of PD. Hypertension is linked to fast dopaminergic neurodegeneration, and motor symptoms were observed in PD. Diastolic inadequacy might be detected as an early indicator of autonomic dysfunction in PD [112].

### 5.5. A Short on Contrast-Based Imaging for ORGAN

By injecting a radioactive tracer, [121] meta-iodo-benzyl-guanidine, into the sympathetic nervous system of the human heart, it is feasible to visualize it (MIBG) [216]. The innovation of this technique, known as MIBG cardiac imaging, holds a lot of promise as a test to confirm the diagnosis of PD (a state in which MIBG detection in the heart is diminished or absent), to identify those who are at risk of developing PD in the future, and to differentiate PD from related disorders [217]. MIBG cardiac imaging is still considered an experimental approach for detecting PD and is not currently in use as a diagnostic instrument [218].

In a recent study, the loss of the sympathetic nerves of the heart was chemically caused in monkeys to resemble the alterations seen in PD [219]. The cardiac system was then scanned with a variety of new-generation radioactive tracers that bind to inflammation and oxidative stress indicators [220]. This model system can be used to investigate the molecular changes that occur when the sympathetic nerves of the heart are lost, as well as to follow the cardiac system’s response to treatments.

### 5.6. A Short Note on the Effect of COVID-19 Infection on PD

Exacerbations of parkinsonian symptoms are frequently caused by COVID-19 infections [221]. Although increased cerebral dopamine metabolism, pharmacologic alterations, and direct effects of endotoxins have been observed, the mechanism for this remains unknown [222]. Motor impairment may remain beyond this phase of systemic inflammation, even if it is typically reversible. PD patients having COVID-19 is a severe illness that has a direct negative impact on PD motor symptoms [223]. According to one research, motor and non-motor symptoms worsened in PD patients infected with COVID-19, either before or after infection. Furthermore, indirect effects such as social isolation, pharmacologic effects, abrupt shifts in schedule, the influence of fear and depression, and continuous lack of mobility are all likely to have negative effects on motor and non-motor symptoms as well as the quality of life in people with PD [2].

### 5.7. A Short Note on Bias in AI System

Almost 18 million deaths occur every year from CVD/stroke around the world. PD affects about 1% of persons over the age of 60 and 5% of adults over the age of 85 [224]. PD symptoms usually strike people once they reach the age of 60. Early and precise diagnosis of CVD/stroke risk stratification in PD is critical for lowering these fatality rates. As a result, to enhance the prediction of CVD/stroke risk stratification in PD, AI systems were introduced as an alternative to LBBM, OBBM, and MedUSE based existing tools. However, there are certain issues with AI systems, since they sometimes focus solely on accuracy, neglecting clinical and scientific validation [75], not matching the gold standard and ground truth, and inaccurately calculating the disease severity ratio. It overemphasizes AI system accuracy while underemphasizing AI system validity. It makes the AI system to be biased [225]. It is also important to note that the database contains particular geographical patient characteristics; as a consequence, the model may provide inaccurate test findings for various continents [226]. As a result, it is critical to identify each AI system’s bias in addition to enhancing CVD/stroke risk stratification in PD [190].

### 5.8. Strengths, Weakness, and Extensions

We provide further help to the existing healthcare systems by establishing the link between PD with CVD and stroke. Prevention is indeed preferable to treatment. With awareness of the link between PD with CVD and stroke, as well as low-cost screening utilizing AI-based algorithms, patients can be not only treated but also prevented from developing the complicated condition. One constraint we see is that neither protocol has been built for treating PD patients with CVD and stroke as covariates, and it is critical to shedding more light on this. Still, there is no clear hypothesis in the AI system to predict the risk of CVD and stroke risk stratification in PD disease, but many AI models solve the problem of diagnosis of the CVD, stroke, and PD diseases separately. The unavailability of the multi-center data on PD with CVD and stroke as comorbidity is also a challenge. With the continuing pandemic, it is critical to consider how the SARS-CoV-2 virus might affect both of the targeted diseases. In the future, we anticipate more systematic reviews on PD-based RoB with comorbidities focusing on the SARS-CoV-2 virus, CVD, and stroke. In addition, in the future, we would like to express how the role of big data is important to understand for minimizing the generational bias in AI models.

## 6. Conclusions

The importance of CVD and stroke risk stratification in PD patients were discussed in this systematic review. We also demonstrated how PD complications can lead to vascular stroke and cerebral stroke. The concept that PD might aggravate CVD and stroke was underlined in this review. As a result, detecting CVD problems in PD patients is critical. Carotid imaging was also shown to be a low-cost, non-invasive alternative to conventional imaging modalities for CVD screening in PD patients. This low-cost B-mode ultrasonography will also be useful for the characterization of plaque tissue in PD patients, providing a crucial additional understanding of CVD and stroke risk stratification in PD patients. Furthermore, we demonstrated that AI-based methods are effective in predicting CVD and stroke risk stratification in PD patients. The AI-based feasible model for CVD and stroke stratification in PD patients was presented. Finally, we comment on the roles of PD with CVD and stroke in the COVID-19 paradigm, as well as the function of AI in this framework.

## Figures and Tables

**Table 1:** The studies show the relation between Parkinson’s and Cardiovascular disease.

SN	Citations	Relation *	ME	PS	Outcome	TRE
1	Cuenca-Bermejo et al. [105] (2021)	Cardiac changes in PD	LBBM	NR	In PD patients with a lack of sympathetic innervation in the heart, cardiac abnormalities have also been identified. Post-prandial hypotension, supine hypertension, increasing blood pressure variability, reduced heart rate variability, and chronotropic incompetence are also symptoms.	NR
2	Park et al. [106] (2020)	PD with risk of CVD	Population-based cohort study	NR	PD was linked to an increased risk of cardiovascular disease. Physicians must also pay attention to CVD prevention in individuals with PD.	NR
3	Potashkin et al. [92] (2020)	Relation between CVD and PD	LBBM	47	Inflammation, insulin resistance, lipid metabolism, and oxidative stress are among the basic mechanisms that both CV disease and PD share. Physical exercise and moderate coffee intake are two modifiable risk variables that are inversely related to both CV disease and PD.	NR
4	Değirmenci et al. [83] (2020)	Cardiac effect of PD	LBBM	NR	Cardiac problems are frequent in PD patients. PD is associated with CVD, such as coronary artery disease, heart failure, cardiac autonomic dysfunction, heart failure, sudden death, and hypertension.	Levodopa, Monoamine oxidase B inhibitors, catechol-O-methyl transferase inhibitors, anticholinergic drugs, deep brain simulations
5	Fanciulli et al. [91] (2020)	Orthostatic hypertension in PD	LBBM	NR	Syncope, unexplained falls, lightheadedness, cognitive impairment, blurred vision, dyspnea, weariness, and shoulders, neck, or low-back discomfort are all symptoms of Orthostatic hypotension. They appear when you stand up and go away when you lie down.	Droxidopa, fludrocortisone, clonidine, transdermal nitroglycerin, nifedipine
6	Yan et al. [107] (2019)	Relation of Carotid plaque in PD	LBBM	68	As PD becoming worsening, the thickness of carotid plaques also increases.	NR
7	Scorza et al. [108] (2018)	Cardiac abnormalities in PD	LBBM	NR	Cardiovascular autonomic dysfunction, cardiomyopathy, coronary heart disease, arrhythmias, conduction abnormalities, and sudden cardiac death are all symptoms of PD/PS.	NR
8	Günaydın et al. [85] (2016)	CVD risk in PD under levodopa treatment	LBBM	65	Compared to healthy people, those with PD who use L-dopa have increased aortic stiffness and poor diastolic performance. Homocysteine levels in the blood may be a potential pathophysiological factor.	NR
9	Huang et al. [92] (2015)	plasma cholesterol risk in PD	LBBM	156	Statin usage has been linked to an increased risk of PD, although larger total cholesterol has been linked to a decreased risk.	Statins
10	Vikdahl et al. [109] (2015)	CVD risk in PD	LBBM	147	High blood cholesterol levels, smoking habits, and a high body mass index (BMI) have all been considered risk factors for PD. A moderate degree of physical exercise may help to lower the risk of heart disease.	NR
11	Goldstein [47] (2014)	Dystonia in PD	LBBM	23	Orthostatic hypotension in PD can be explained by the loss of sympathetic nerves and the associated failure of the baroreflex. During levodopa medication, hypotension might exacerbate after standing or after a substantial meal.	NR
12	Liang et al. [31] (2015)	Risk of CAD due to PD	LBBM	NR	PD is related to an increased risk of AMI; the mechanism needs to be explained.	NR
13	Goldstein [110] (2014)	Cardiac denervation in PD	LBBM	40	In individuals with PD and neurogenic orthostatic hypotension, cardiac sympathetic denervation is almost ubiquitous. Before the start of the movement disorder, baroreflex-cardiovagal failure and cardiac sympathetic denervation can occur, suggesting that neuroradiologic testing might be used as a biomarker for diagnosing presymptomatic or early PD and monitoring responses to possible neuroprotective therapies.	NR
14	Pan et al. [111] (2013)	Relation between Serum Uric acid with vascular PD	LBBM	160	Low uric acid levels are more likely to develop PD, and the inverse connection between uric acid and PD severity was strong for males but weak for women. There is no connection for uric acid found in vascular PD.	NR
15	Wong et al. [97] (2012)	PD with Cardiac Sympathetic Denervation	LBBM	27	In IPD, there is a sign of cardiac sympathetic denervation.	NR
16	Czarkowska et al. [112] (2010)	PD with Cardiac response	LBBM	53	With the progression of PD, cardiac responses to orthostatic stress worsen. The fall is caused by the detonation.	NR
17	Buob et al. [113] (2010)	Cardiac dysfunction in PD	LBBM	07	The chronotropic and contractile responses mediated by catecholamines rule out a functionally significant sympathetic malfunction. Sympathetic denervation maybe still not be complete, and the surviving fibers are enough to sustain autonomic control.	NR
18	Walter et al. [114] (2008)	PD with Cardiovascular autonomic dysfunction	LBBM	NR	Other parkinsonian illnesses are characterized by peripheral autonomic dysfunction.	Somatostatin, levodopa

SN: serial number; * RELATION: Effect of PD on CVD; ME: method of evaluation; PS: patient size; TRE: treatment; NR: not reported; AMI: acute myocardial Interaction; LBBM: laboratory based biomarkers.

**Table 2:** The studies show the relationship between Parkinson’s and stroke.

SN	Citations	Relation	ME	PS	Outcome	TRE
1	Li et al. [140] (2018)	Stroke and CAD in PD	LBBM	63	Stroke risk was observed to be higher in people with PD. Cerebral small vessel disease has been linked to moderate parkinsonian symptoms.	NR
2	Studer et al. [90] (2017)	Heart rate variability and skin resonance in PD	LBBM	73	Both SSR and HRV measurements are sensitive in diagnosing ANS dysfunction, not only in the late stages of PD but also in the early stages and can be used to diagnose autonomic derangement in PD patients.	NR
3	Liu et al. [11] (2014)	Stroke in PD	Self-reporting a specialist for the diagnosis	32	Cerebral infarction is intimately linked to PD due to cerebrovascular and neurodegenerative disorders coincide. Although levodopa causes OH and raised homocysteine, which may increase the risk of stroke, it remains the most effective and essential symptomatic therapy for many people with PD.	NR
4	Becker et al. [18] (2009)	Risk of stroke in PD	LBBM	NR	Hyperhomocysteinemia might be a relationship between PD and an increased risk of ischemic stroke. Homocysteine levels beyond a certain threshold have been proven to increase the risk of stroke and coronary artery disease. vascular disease and dementia, as well as the fact that levodopa treatment is linked to both with a rise in homocysteine in the blood.	NR
5	Levine et al. [141] (2009)	Traumatic brain injury in PD	LBBM	NR	A potential technique for reducing both physical and cognitive weariness in people with neurologic diseases is exercise training. In people with PD, a cardiovascular exercise plan can help to reduce overall weariness.	NR
6	Rickards [142] (2005)	Stroke in PD	NR	NR	Depressive syndromes in chronic neurological illnesses are common and disabling. Their etiology is complex and may be multifactorial in individual patients.	NR
7	Mastaglia et al. [143] (2002)	Prevalence stroke in PD	Self-reporting a specialist for the diagnosis	100	Postmortem investigation, studies did not directly compare our findings to other studies of stroke-related mortality and morbidity in the PD population.	NR

SN: serial number; RELATION: Effect of PD on Stroke; ME: method of evaluation; PS: patient size; TRE: Treatment; NR: not reported; SSR: sympathetic skin response; HRV: heart rate variability; OH: orthostatic hypotension; LBBM: laboratory based biomarkers.

**Table 3:** The table shows the prediction of CVD by using AI.

SN	Citations	IC	DS	GT	FE	TOC	ML vs. DL	ACC %	AUC
1	Suri et al. [189] (2022)	OBBM, CUSIP	117	CVD, Bias	NR	NR	ML	NR	NR
2	Kandha et al. [190] (2020)	OBBM, LBBM	346	Death	DCNN	NB, SVM, KNN, DT	DL	83.33	0.833
3	Jamthikar et al. [30] (2020)	OBBM, LBBM, CUSIP	202	CVD	SVM	NR	ML	92.53	0.92
4	Skandha et al. [191] (2020)	OBBM, LBBM	246	Stroke	11 Models	NR	HDL	98.30	0.983
5	Saba et al. [192] (2020)	OBBM, LBBM, CUSIP	246	Death	6 Models	NR	HDL	89.00	0.898
6	Jamthikar et al. [177] (2019)	OBBM, LBBM (US)	395	CVD	PCA	RF	ML	95.00	0.80
7	Biswas et al. [193] (2018)	OBBM, LBBM (US)	407	Stroke, Diabetes	NR	CNN	DL	99.61	0.99

SN: serial number, IC: input covariates, DS: data size, GT: ground truth, OBBM: office base biomarker, LBBM: laboratory based biomarkers, FE: feature extraction, TOC: type of classifier, ACC: percentage accuracy, US: ultrasound, NR: not reported.

**Table 4:** The table shows the prediction of stroke by using AI.

SN	Citations	IC	DS	GT	FE	TOC	ML vs. DL	ACC %	AUC
1	Soun et al. [194] (2021)	LBBM (CT)	209	Stroke	NN	AlexNet	DL	96.09	0.96
2	Reva et al. [195] (2021)	OBBM, LBBM	200	Stroke, CT	NB	DT, RF, SVM	ML	85.32	NR
3	Murray et al. [9] (2020)	OBBM, LBBM	341	LVO, Stroke	RF	CNN	HDL	85.00	NR
4	Mouridsen et al. [196] (2020)	OBBM, LBBM, CUSIP	16	Stroke, MRI	NR	CNN	DL	74.00	0.74
5	Yu et al. [147] (2020)	OBBM, LBBM (EMG)	287	Stroke, EMG	SVM	RF, LSTM	ML	98.33	0.98
6	Ain et al. [197] (2020)	OBBM, LBBM	130	Stroke, non-stroke	NB	NB	ML	84.00	NR
7	Badriyah et al. [198] (2020)	OBBM (CT)	29	Stroke	NB	DT, RF, SVM	HDL	94.30	NR

SN: serial number, IC: input covariates, DS: data size, GT: Gground truth, OBBM: office-based biomarker, LBBM: laboratory based biomarkers, FE: feature extraction, TOC: type of classifier, ACC: percentage accuracy, CT: computer tomography, EMG: electromyography, MRI: magnetic resonance imagining, NR: not reported.

**Table 5:** The table shows the prediction of Parkinson’s by using AI.

SN	Citations	IC	DS	GT	FE	TOC	ML vs. DL	ACC %	AUC
1	Bikias et al. [199] (2021)	LBBM (FoG)	18	PD vs. Non PD	SVM	CNN	DL	90.00	NR
2	Pramanik et al. [200] (2021)	LBBM (Voice)	252	PD vs. Non PD	NB	RF	ML	95.00	NR
3	Borzì et al. [201] (2021)	OBBM, LBBM (FoG)	11	PD vs. Non PD	RF	NB	ML	84.10	NR
4	Aich et al. [202] (2020)	OBBM, LBBM (FoG)	20	PD vs. Non PD	RF	SVM, RF, KNN	ML	97.35	0.74
5	Pramanik et al. [203] (2021)	LBBM (Voice)	169	PD vs. Non PD	NB	SVM, RF	ML	78.97	0.78
6	Zahid et al. [204] (2020)	LBBM (Voice)	50	PD vs. Non PD	SVM	RF	HDL	99.1	NR
7	Nissar et al. [205] (2019)	LBBM (Voice)	188	PD vs. Non PD	NB	XGBoost	ML	92.76	NR

SN: serial number, IC: input covariates, DS: data size, GT: ground truth, OBBM: office-based biomarker, LBBM: laboratory based biomarkers, FE: feature extraction, TOC: type of classifier, ACC: percentage accuracy, AUC: Area Under Curve, FoG: freezing of gait, NR: not reported.

**Table 6:** Comparative analysis of studies CVD and stroke risk stratification in PD Patient. Y: yes, N: no, PD: Parkinson’s disease, CVD: cardiovascular Disease, AI: artificial Intelligence.

SN	Citations	Year	PD	CVD	Stroke	AI	COVID-19
1	Li et al. [70]	2018	Y	N	Y	N	N
2	Jamthikar et al. [18]	2020	N	Y	N	Y	N
3	Mouridsen et al. [122]	2020	N	N	Y	Y	N
4	Bikias et al. [119]	2021	Y	N	N	Y	N
5	Reva et al. [120]	2021	N	N	Y	Y	N
6	Bermejo et al. [72]	2021	Y	Y	N	N	N
7	Pramanik et al. [121]	2021	Y	N	N	Y	N
8	Suri et al. (Proposed)	2022	Y	Y	Y	Y	Y

## Glossary

Acute Stroke	A stage of stroke that starts at the beginning of symptoms and lasts for a few hours after.
ANOVA	Is an analysis tool used in statistics that splits an observed aggregate variability found inside a dataset into two parts: systematic factors and random factors.
Arrhythmia	An abnormal heartbeat.
Arteriosclerosis	A disease process, commonly called “hardening of the arteries,” includes a variety of conditions that cause artery walls to thicken and lose elasticity.
Artificial Intelligence	Artificial intelligence (AI) is intelligence demonstrated by machines, as opposed to the natural intelligence displayed by animals, including humans.
Atherosclerosis	A disease in which plaque builds up inside your arteries. This narrows the arteries and blocks blood flow to the brain, which increases the risk of a stroke.
Autonomic nervous system	The part of the body’s complex system of nerves that controls the involuntary activity of some of the internal organs, such as breathing or heartbeat.
Basal ganglia	These are structures located deep in the brain that are responsible for normal movement, such as walking. The basal ganglia are made up of three main parts, the caudate nucleus, the putamen, and the globus pallidus.
Bradycardia	Abnormally slow heartbeat.
Bradykinesia	Slowing down of movement. It is a major symptom of Parkinson’s.
Cardiac arrest	The stopping of the heartbeat, usually because of interference with the electrical signal.
Cardiovascular	About the heart and blood vessels that make up the circulatory system.
Carotid artery	An artery located on either side of the neck supplies the front part of the brain with blood.
Cerebellum	Part of the brain is involved in the coordination of movements.
Cerebral cortex	The largest part of the brain is responsible for thought, reasoning, memory, sensation, and voluntary movement.
Cerebrovascular Disease	One or more diseases are caused by blood flow (circulation) problems, such as blood flow restriction or a blockage or clot, in vessels that supply blood to the brain.
Chorea	A type of abnormal movement or dyskinesia, characterized by continuing, rapid, dance-like movements. May result from high doses of levodopa and/or long-term levodopa treatment.
Cognitive Impairment	Difficulty with thinking abilities such as paying attention, memory, communication, and problem-solving.
Cogwheel rigidity	Stiffness in the muscles, with a jerky quality, when arms and legs are repeatedly moved.
Congestive heart failure	A condition in which the heart cannot pump all the blood returning to it, leading to a backup of blood in the vessels and an accumulation of fluid in the body’s tissues, including the lungs.
Deep Learning	Are a type of machine learning and artificial intelligence (AI) that imitates the way humans gain certain types of knowledge.
Dementia	The loss of some intellectual abilities is characterized by a loss of awareness and confusion.
Dopamine	A chemical produced by the brain; assists in the effective transmission of messages from one nerve cell to the next. People with Parkinson’s have decreased amounts of the chemical in the basal ganglia and substantia nigra, two structures located deep in the brain. Dopamine coordinates the actions of movement, balance, and walking.
DVT (Deep Vein Thrombosis)	A blood clot that forms in a vein deep in the body. It can cause a potentially life-threatening complication if the clot detaches and moves to the lungs resulting in a blockage known as a pulmonary embolism (PE).
Dysarthria	Difficulty saying words clearly due to problems with muscle strength and coordination.
Dysarthria	Speech difficulties due to impairment of the muscles associated with speech.
Dyskinesia	Abnormal muscle movements. These may appear as a side effect of long-term drug treatment in Parkinson’s and may worsen in response to stress.
Dysphagia	Difficulty with swallowing.
Edema	Swelling is caused by fluid accumulation in body tissues.
Embolic Stroke	A stroke is caused by an embolus (a free-floating mass traveling through the bloodstream). The embolus may be a blood clot (thrombus), a ball of fat, a bubble of air or other gas (gas embolism), or foreign material.
Hemorrhagic Stroke	Sudden bleeding into or around the brain. It is also called a brain hemorrhage or brain bleed.
Heredity	The genetic transmission of a particular quality or trait from parent to child.
High-density lipoprotein (HDL)	Also known as “good cholesterol.” HDL helps move the “bad cholesterol” from the arteries back to the liver; thus, it can break down and leave the body.
Hypertrophy	Enlargement of tissues or organs because of increased workload.
Hypoxia	A state of decreased oxygen delivery to a cell thus that the oxygen falls below normal levels.
Intracerebral Hemorrhage (ICH)	A type of stroke occurs when a vessel within the brain leaks blood into the brain.
Ischemic Stroke	Damage to the brain is caused by a lack of blood flow, usually from a clot.
Levodopa	A drug containing a form of the important brain chemical dopamine commonly used to treat symptoms of Parkinson’s disease. In combination with carbidopa, it is called Sinemet; combined with benserazide, it is called Prolopa.
Lewy body	Brain cells have abnormally pigmented spheres inside them. They are found in the damaged parts of the brain in people with Parkinson’s disease.
Low-density lipoprotein (LDL)	Also known as the “bad cholesterol”; a compound that carries most of the total cholesterol in the blood and deposits the excess along the inside of arterial walls.
Machine learning	Machine learning is a method of data analysis that automates analytical model building.
Myocardial infarction	A heart attack. The damage or death of an area of the heart muscle (myocardium) resulting from a blocked blood supply to the area. The affected tissue dies, injuring the heart. Symptoms include prolonged, intensive chest pain, and a decrease in blood pressure that often causes shock.
Navi byes	Naive Bayes classifiers are a family of simple “probabilistic classifiers” based on applying Bayes’ theorem with strong (naive) independence assumptions between the features.
Principal Component Analysis	Is the process of computing the principal components and using them to perform a change of basis on the data, sometimes using only the first few principal components and ignoring the rest.
Pulmonary Embolism (PE)	A blockage of an artery in the lungs by a substance that has traveled from elsewhere in the body through the bloodstream. Severe cases can lead to passing out, abnormally low blood pressure, and sudden death.
Random forests	Is an ensemble learning method for classification, regression, and other tasks that operates by constructing a multitude of decision trees at training time?
Resting tremor	Shaking occurs in a relaxed and supported limb.
Rigidity	Muscular stiffness is common in people with Parkinson’s disease. It is characterized by a resistance to movement in the limbs.
Stenosis	Narrowing of an artery due to the buildup of plaque within the artery.
Stroke	Occurs when the blood supply to part of the brain is suddenly interrupted or when a blood vessel in the brain bursts, spilling blood into the spaces surrounding brain cells. There are two types of stroke: ischemic (clot) or hemorrhagic (bleeding).
Support Vector Machine	Supervised learning models with associated learning algorithms that analyze data for classification and regression analysis.
Thrombosis	The formation of a blood clot in one of the brain arteries of the head or neck that stays attached to the artery wall until it grows large enough to block blood flow.
**Abbreviations**	
ACC	American College of Cardiology
AHA	American Heart Association
ANOVA	Analysis of variance
ASCVD	Atherosclerotic cardiovascular disease
ANS	Autonomic Nervous System
AUC	Area-under-the-curve
AI	Artificial Intelligence
BMI	Body mass index
CAD	Coronary artery disease
CAS	Coronary artery syndrome
CHD	Coronary Heart Disease
CKD	Chronic kidney disease
CT	Computed Tomography
CUSIP	Carotid ultrasound image phenotype
CV	Cross-validation
CVD	Cardiovascular disease
CVE	Cardiovascular events
DA	Endogenous Dopamine
DL	Deep learning
DM	Diabetes mellitus
EEGS	Event-equivalent gold standard
EMG	Electromyography
FH	Family history
FoG	Freezing of Gait
GT	Ground truth
HTN	Hypertension
HDL	Hybrid deep learning
ICAM	Intercellular Adhesion Molecule
VCAM	vascular cell adhesion molecule
LBBM	Laboratory-based biomarker
MedUSE	Medication use
ML	Machine learning
MRI	Magnetic Resonance Imaging
MIBG	Iodine-123 meta-iodobenzylguanidine
NPV	Negative predictive value
NB	Naive byes
NO	Nitric Oxide
nOH	Neurogenic orthostatic hypotension
Non-ML	Non-machine learning
OBBM	Office-based biomarker
OH	orthostatic hypotension
OxLDL	Oxidation of low-density lipoprotein
QTc	chaotic heartbeat
PD	Parkinson Disease
PE	Performance evaluation matrices
PPV	Positive predictive value
PCA	Principal Component Analysis
PTC	Plaque tissue characterization
RA	Rheumatoid arthritis
PR	Period measured in milliseconds
RF	Random forest
ROS	Reactive Oxides Stress
RoB	Risk of bias
ROC	Receiver operating-characteristics
SCORE	Systematic coronary risk evaluation
SMOTE	Synthetic minority over-sampling technique
SVM	Support vector machine
TPA	Total plaque area
US	Ultrasound
DNA	Deoxyribonucleic acid
